# Supplementary material for: Chromothripsis during telomere crisis is independent of NHEJ, and consistent with a replicative origin
Source: Genome Res. 2019 May;29(5):737–49. doi: 10.1101/gr.240705.118 (PMC6499312; doi:10.1101/gr.240705.118)
Supplement: Supplemental Material [file supp_gr.240705.118_Supplemental_file_1.zip › contigs/annotated_contigs/DB51/contig.2.DB51_length_670_mean_cov_10.9701492537.docx]

**DB51_length_670_mean_cov_10.9701492537**

```
GAGTGAGTGAGTGTGGAAGGAACATCTGAGGATTGTCCCCAAGAGGGATGGGACTGGCAAAATGTCACTGGCATTGGAAGTAGGAAGCA
 >chr6:7798569-7798892 - E=1e-173 p=2e-02
GACTGTTGGGAACAGGTAGAACACGAACCCTAAATTAGTGTGGTTTTGCTCATTTGCAAAAATGATGTGAGATACTTGCTGAGATGCTC

ATGCGCACTGTATCAGAAACAGTGCTGGACTGGAAGGGTGGTCACCCAGAGCCAACTCTAAGGGGCTGCATGACTGCGAACAGGGACTT

GACCTCTTTTGGCTTCAGTTCACCATCAGTAAGGTGAGGAAAGTGGTCCAGACCA|T|ATTCTGTTCTATTGGCCTACATGACTGCTTT
                                                        >chr6:81124314-81124662 + E=1e-
TATACCAGCACCACGTTGTTTTATTTACTGTAGCCTTGTACAATTTTAAGTCAGGTAACTTGATTTCCAGCCGTGTTCTTTTTGATTAG
197
GATTGACTTGGCTATTGGGGCTCTTTATGGTTCCATAGAAATTTTAAAATAGCTTTTTCCTAGTTCTGTAAAAAATGCCAATTGTAGTT

TAATAGGAGTAGCATTGAACCTTTAAATTGCTTTGGGCTGTTTGGACATTACAAGGATATTGAATCTTCCTATTCGGGAGCATGGAATG

TTTTTTCATTGGTTTGTGTCATTCCTGATTTCTTTGAACAATGTTTTGT
```
